# A transgenic mouse line for rabies virus-mediated trans-synaptic tracing in the postnatal developing brain

**DOI:** 10.1371/journal.pone.0323629

**Published:** 2025-05-12

**Authors:** Kengo Inada, Mitsue Hagihara, Miho Kihara, Takaya Abe, Kazunari Miyamichi

**Affiliations:** 1 Laboratory for Comparative Connectomics, RIKEN Center for Biosystems Dynamics Research, Kobe, Hyogo, Japan; 2 Laboratory for Animal Resources and Genetic Engineering, RIKEN Center for Biosystems Dynamics Research, Kobe, Hyogo, Japan; Johns Hopkins University, UNITED STATES OF AMERICA

## Abstract

Neural circuits are composed of numerous neurons that perform diverse functions. Understanding the mechanisms of neural processing requires elucidating the connections among individual neurons. Rabies virus (RV)-mediated trans-synaptic tracing enables the visualization of direct presynaptic neurons of a defined neural population, facilitating the precise mapping of neural circuits across various brain regions. This method relies on RV mutants that require the expression of the TVA receptor and rabies glycoprotein to infect and spread to presynaptic neurons. Traditionally, adeno-associated virus (AAV) has been used to express these proteins. However, because AAV requires several weeks to achieve sufficient gene expression, it is challenging to use this approach for studying neural connections during postnatal development. To address this limitation, we generated a transgenic mouse line, termed *Ai162-nCTG*, which expresses nuclear-localized mCherry, the TVA receptor, and rabies glycoprotein in a Cre-dependent manner. As a proof-of-principle, we crossed the *Ai162-nCTG* line with the *vasopressin-Cre* line. In the paraventricular hypothalamic nucleus, where a major cluster of vasopressin neurons exists, mCherry expression was highly specific to vasopressin neurons, although not all vasopressin neurons co-expressed mCherry. We injected RV into the paraventricular hypothalamic nucleus and compared the labeling patterns with those of the conventional AAV-based approach. Although both methods labeled input cells in similar brain regions, the AAV-based approach was superior in terms of labeling efficiency. We also demonstrated that the *Ai162-nCTG*-based method enables rabies virus-mediated trans-synaptic tracing in mice at postnatal day 7 and 30. The distribution of presynaptic neurons was largely similar in the juvenile and adult stages, suggesting that paraventricular vasopressin neurons do not significantly change their presynaptic inputs during post-weaning development. Taken together, these findings suggest that the *Ai162-nCTG* line can be used for rabies virus-mediated trans-synaptic tracing when AAV administration is challenging. We also acknowledge and discuss the technical constraints associated with this mouse line.

## Introduction

Neural circuits organize numerous neurons to exert specific functions. Identifying neural connections between neurons provides fundamental knowledge for a more complete understanding of neural processing [1,2]. Over the past decades, viral methods have been developed to visualize neural connections in the mouse brain [3,4]. Among these methods, RV-mediated trans-synaptic tracing, which visualizes direct presynaptic neurons of a defined population of neurons, is widely used for analyzing neural connections [5,6]. This method involves a two-step serial modification of an attenuated rabies strain called Street Alabama Dufferin (SAD) B19. First, an essential rabies gene encoding rabies glycoprotein (RG) is removed and replaced with the *EGFP* gene, resulting in SAD*ΔG-EGFP* [7]. Second, the tropism of SAD*ΔG-EGFP* is altered by pseudotyping with the envelope protein of avian sarcoma and leucosis virus, known as EnvA [8], which restricts viral infection to cells expressing the corresponding receptor, TVA, a protein found in birds but not in mammals [9]. The resulting RV mutant, referred to as SAD*ΔG-EGFP*+EnvA, can infect genetically modified target neurons where the expression of *RG* and *TVA* transgenes has been introduced. These initially infected neurons that express both RG and TVA receptor are referred to as ‘starter’ cells, from which, the retrograde trans-synaptic spread of RV occurs for only a single synaptic step [8,10].

Typically, cell type-specific expression of the TVA receptor and RG has been achieved by injecting a mixture of Cre-dependent AAVs that express each [11,12], or an AAV that expresses both [10,13]. Despite its versatility, this AAV-based expression method has two major drawbacks. First, it requires several weeks to achieve sufficient levels of TVA receptor and RG expression, making it challenging to perform rabies virus-mediated trans-synaptic tracing in juveniles or neonates. Second, the same region must be injected twice, several weeks apart, to initiate tracing, which can be challenging in organs difficult to target with AAV. Genetic expression of the TVA receptor and RG using a knock-in mouse is an alternative approach. A previous study generated a mouse line expressing the TVA receptor and RG in a Cre-dependent manner [14]. However, this line does not co-express fluorescent molecules, which makes it difficult to visualize starter cells. Additionally, the simple *CAG* promoter-based single-copy transgene may limit the expression levels of RG, potentially compromising the efficiency of trans-synaptic tracing.

Recent advances in mouse genetics have provided a novel expression system that allows high-level expression from a single-copy transgene, comparable to that achieved by AAV vectors [15]. Here, we report a new transgenic mouse line, named *Ai162-nCTG*, which expresses nuclear-localized mCherry (nuc-mCherry), the TVA receptor, and RG from a modified *Ai162* allele in a Cre-dependent manner. We introduced the *TIGRE2.0* transgenic platform [15] to achieve sufficiently high-level expression of the effector genes. *Ai162-nCTG* facilitates rabies virus-mediated trans-synaptic tracing during postnatal development, albeit with several experimental constraints and limitations, as described below.

## Results

### Generation and characterization of the *Ai162-nCTG* mouse line

**Fig 1:**
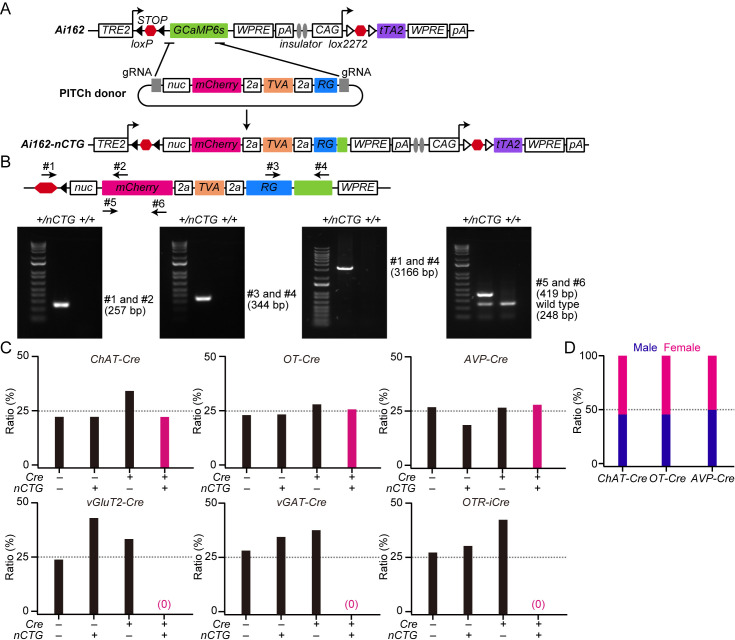
Design of the *Ai162-nCTG* mouse line and its transmission to progeny. (A) Schematic representation of the procedure used to generate *Ai162-nCTG* using the PITCh system. *Ai162-nCTG* expresses nuclear-localized mCherry (nuc-mCherry), the TVA receptor, and RG in a Cre-dependent manner. (B) Top: Schematic showing the targets of genotyping primers. Bottom: Representative results of gel electrophoresis. (C) Ratio of each mouse genotype. Males heterozygous for *Cre* (+/*Cre*) and females heterozygous for *nCTG* (*+/nCTG*) were crossed. The dotted line indicates the expected Mendelian ratio. n = 6 mothers for *ChAT-Cre*, *OT-Cre*, *vGluT2-Cre*, *vGAT-Cre*, and *OTR-iCre*. n = 12 mothers for *AVP-Cre*. (D) Ratio of males and females for the mice harboring both *Cre* and *nCTG* shown in (C). The dotted line indicates the expected ratio.

To generate a mouse line expressing the nuc-mCherry, the TVA receptor, and RG in a Cre-dependent manner, we obtained the *Ai162* mouse line, which expresses GCaMP6s based on the *TIGRE2.0* approach [15]. We then sought to replace the *GCaMP6s* gene in the *Ai162* mouse genome with *nuc-mCherry*, followed by the *TVA* and *RG* genes linked by self-cleaving 2a peptides, using the CRISPR-Cas9-based precise integration into the target chromosome (PITCh) system [16] (Fig 1A; Materials and Methods). In the presence of Cre, STOP signals are excised, which allows the *CAG* promoter to drive tTA2 transactivator expression. This tTA2, in turn, activates the expression of the effector genes from the *TRE2* promoter (Fig 1A; Materials and Methods).

A mixture of CRISPR RNAs, the PITCh donor vector, and Cas9 protein was microinjected into the pronuclei of *Ai162* one-cell stage zygotes. We obtained 77 F_0_ founder mice, 16 of which were positive for the *mCherry* gene in the primary PCR screening. Of these, two had sequence-confirmed junction structures where the knock-in had occurred. Two mouse lines, designated *Ai162-nCTG* (or *nCTG* for short) Lines 1 and 2, were established from these founders. Subsequent PCR-based analysis of the integrity of the knock-in cassette (Fig 1B) revealed that Line 1 showed an inverted duplication of portions of the knock-in cassette, rendering it nonfunctional. Line 2, on the other hand, displayed the expected PCR band lengths in gel electrophoresis with each primer combination (Fig 1B and S1–S3 Figs). Hereafter, we solely describe the results obtained using *Ai162-nCTG* Line 2.

It is known that certain combinations of a Cre driver and a *TIGRE2.0*-based *Ai* reporter mouse line can lead to embryonic lethality or abnormal growth in mice [15]. Given that our *Ai162-nCTG* line relies on the *TIGRE2.0* system, we examined its versatility. We crossed heterozygous females of this line with heterozygous males of several Cre lines (Fig 1C). According to Mendel’s law, four types of genotypes should appear with a probability of 25% in the F_1_ generation. We found that progenies of some Cre lines, specifically those that express Cre in neurons positive for *choline acetyltransferase* (*ChAT-Cre*), *oxytocin* (*OT-Cre*), and *arginine vasopressin* (*AVP-Cre*), largely followed Mendel’s law (Fig 1C). However, from other Cre lines, such as *vGluT2* (*vesicular glutamate transporter type 2*)*-Cre*, *vGAT* (*vesicular GABA transporter*)*-Cre*, and *OTR* (*oxytocin receptor*)*-iCre*, none of the dual-positive progenies were born (Fig 1C). Note that we performed genotyping on postnatal days (P) 5–8, and at that time, dead pups were rarely found. These results suggest the lethality during the embryonic period. This lethality may be similar to that reported for the *TIGRE2.0* system [15] (see Discussion). In *ChAT-Cre*, *OT-Cre*, and *AVP-Cre*, the male-to-female ratio of the mice harboring both *Cre* and *Ai162-nCTG* was approximately 50% (Fig 1D). Collectively, these results suggest that *Ai162-nCTG* enables experiments in both males and females as long as the Cre driver is suitable for the experiment.

### Mice double-positive for *AVP-Cre* and *nCTG* showed reduced levels of *AVP* expression

**Fig 2:**
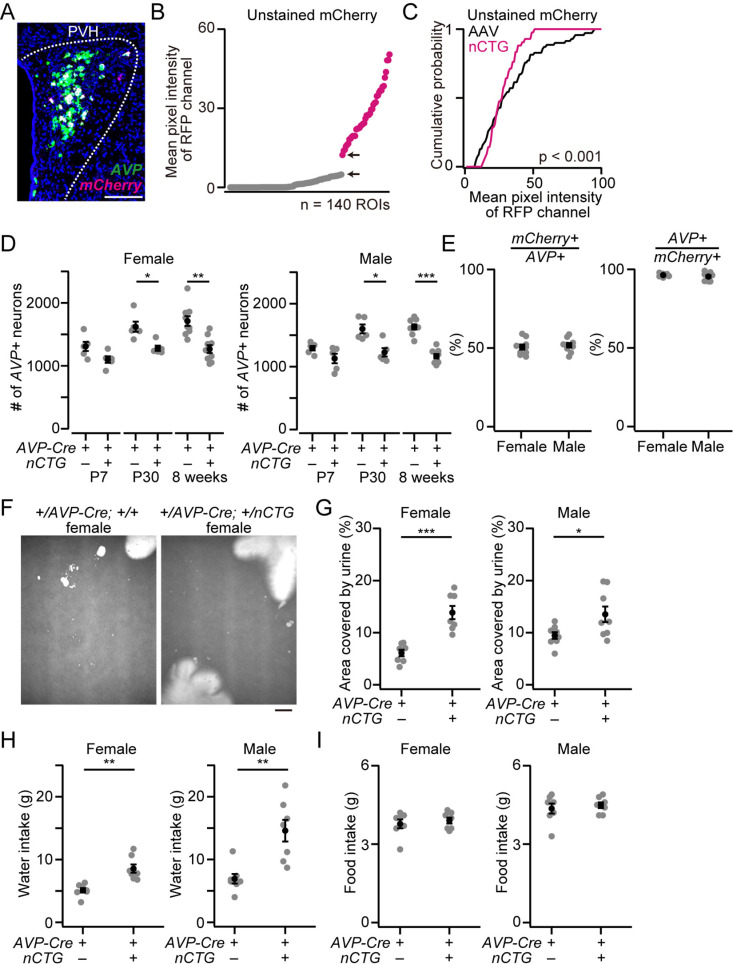
Specificity and efficiency of *Ai162-nCTG* and polydipsia and polyuria exhibited by mice double-positive for *AVP-Cre* and *Ai162-nCTG.* (A) Representative coronal image of the PVH from a double heterozygous mouse harboring *AVP-Cre* and *nCTG*. Green and magenta represent *AVP* mRNA and *mCherry* mRNA, respectively. Blue, DAPI. Scale bar, 50 μm. (B) Distribution of mean pixel intensity of RFP channel in the PVH of +/*AVP-Cre; +/nCTG* mice. Each ROI corresponds to a cell identified by DAPI signal. Note that each brain was analyzed without staining with antibodies. Despite the variability of mCherry expression level, mCherry+ neurons (magenta ROIs) can be identified from their fluorescence (see the interval between the two arrows). n = 140 ROIs from 7 mice. (C) Cumulative probability of mean pixel intensity of RFP channel. “AAV” corresponds to the mice harboring only *AVP-Cre* (+/*AVP-Cre*) that received *AAV-FLEx-TVA-mCherry* (n = 70 ROIs from 7 mice), while “nCTG” was calculated from the data shown as magenta in (B). The p-value is shown in the panel (Kolmogorov–Smirnov test). (D) The number of *AVP*-expressing (*AVP+*) neurons in the PVH was smaller in +/*AVP-Cre; +/nCTG* mice compared with mice harboring only *AVP-Cre* (n = 5 mice each for P7 and P30, n = 9 mice each for 8-weeks-old). Significant difference was found in P30 and 8-week-old mice (*p < 0.05, **p < 0.01, ***p < 0.001, two-tailed Mann-Whitney *U*-test). (E) Approximately half of the *AVP+* neurons were labeled with *mCherry* in +/*AVP-Cre; +/nCTG* mice, while almost all the *mCherry+* neurons co-expressed *AVP* (n = 9 mice each). We did not find significant difference between males and females (two-tailed Mann-Whitney *U*-test). (F) Representative images of the urine-marked area (white). Scale bar, 2 cm. (G) Both male and female +/*AVP-Cre; +/nCTG* mice displayed a larger fraction of the area covered by urine (n = 8 each and 7 each for males and females, respectively; *p < 0.05, ***p < 0.001, two-tailed Welch’s *t*-test). (H) Water intake over 24 h was significan*t*ly larger in +/*AVP-Cre; +/nCTG* mice (n = 7 mice each; **p < 0.01, two-tailed Welch’s *t*-test). (I) Food intake did not show a significant difference (n = 7 mice each). Error bars, SEM.

Because mice harboring both *AVP-Cre* and *Ai162-nCTG* (hereafter, *AVP-Cre; nCTG*) followed the Mendelian ratio, we aimed to provide a proof-of-principle demonstration of trans-synaptic tracing using *AVP-Cre; nCTG* mice. We first performed *in situ* hybridization to visualize *AVP* mRNA and *mCherry* mRNA. In the paraventricular hypothalamic nucleus (PVH), a brain region where *AVP*-expressing (*AVP+*) neurons are known to cluster, we detected neurons expressing *mCherry* (*mCherry+*; Fig 2A). Despite the variability of mCherry expression level, mCherry+ neurons can be clearly identified from their fluorescence (Fig 2B). To compare the fluorescence intensity of *nCTG*-driven mCherry, we injected conventional *AAV5-FLEx-TVA-mCherry* into the PVH of +/*AVP-Cre* (hereafter, *AVP-Cre*) mice. The fluorescence intensity of *nCTG*-driven mCherry expression was within the range of AAV (Fig 2C), suggesting that *nCTG* sufficiently expresses mCherry. We found that the number of *AVP+* neurons in the PVH was significantly smaller in *AVP-Cre; nCTG* mice compared with +/*AVP-Cre* mice in both P30 and 8-week-old mice (Fig 2D). Among those *AVP+* neurons, nearly half were labeled by *in situ* staining for *mCherry* (Fig 2E). We did not find significant difference between males and females (Fig 2E). This relatively low targeting efficiency of nCTG expression may be similar to previous observations of the *TIGRE2.0* system [15]. The specificity of nCTG expression was high, as nearly all *mCherry*+ neurons co-expressed *AVP* (Fig 2E).

During rearing, we noticed that *AVP-Cre; nCTG* mice exhibited increased urine volume, as the wooden chips in their home cages were consistently wetter compared with those of other genotypes, such as *AVP-Cre*. Together with the observation that *AVP-Cre; nCTG* mice possess fewer *AVP*+ neurons in the PVH, we hypothesized that these mice evoked polyuria, presumably accompanied by polydipsia. To examine this possibility, we measured the urine-marking area as a proxy for urine volume and analyzed the water consumption and food intake of *AVP-Cre; nCTG* mice. We found that both the urine-marking area and water consumption were significantly larger in *AVP-Cre; nCTG* mice than in *AVP-Cre* mice, while food intake was not significantly different (Fig 2F–I). Given that AVP contributes to water reabsorption from primitive urine in the kidneys [17], and that a reduction in AVP expression is known to induce polyuria and polydipsia in mice [18,19], these results suggest that *AVP-Cre; nCTG* mice show reduced AVP secretion, likely because of decreased AVP expression in the PVH (Fig 2D) and possibly other brain regions. These findings indicate that *Ai162-nCTG* may induce toxicity in a fraction of Cre-expressing neurons, although whether the observed abnormalities are attributed to the *TIGRE2.0*-based system of the *Ai162* line, the expression of the nCTG cassette, or the combination of both, remains unclear.

### *Ai162-nCTG*-mediated trans-synaptic tracing largely captures the presynaptic landscape

**Fig 3:**
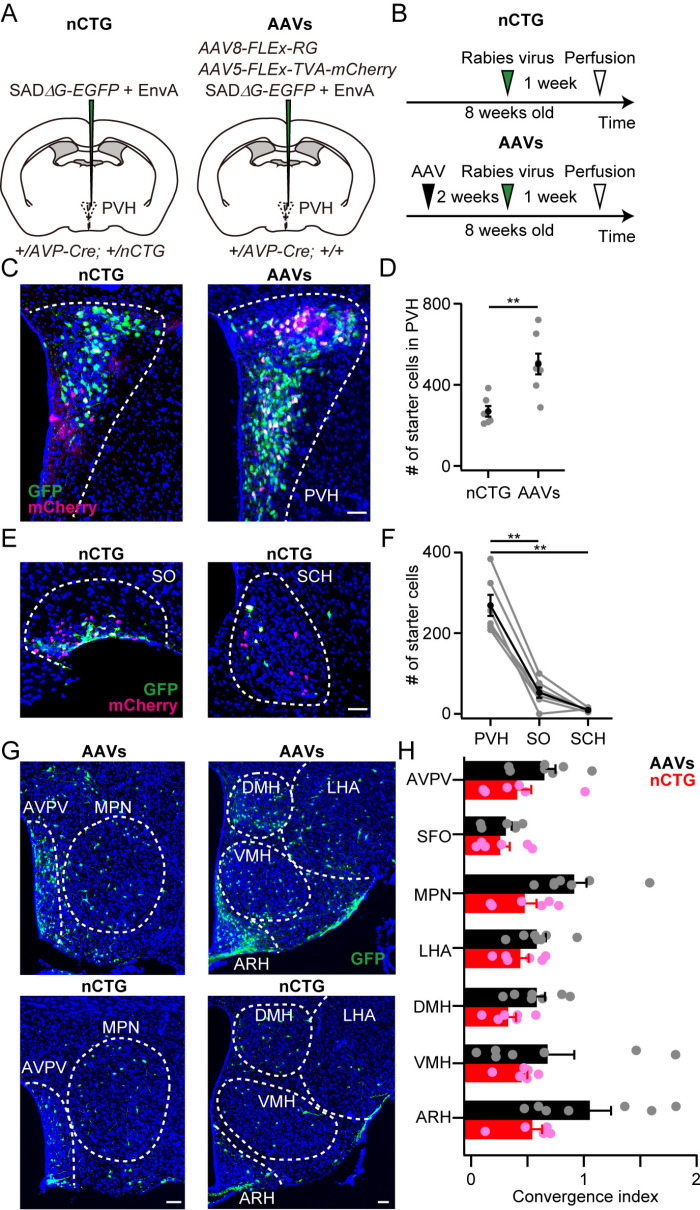
Comparison of the distributions of presynaptic neurons visualized by the *nCTG-* or AAV-based approach. (A, B) Schematic of virus injection (A) and experimental time line (B). Female mice were used in this experiment. Mice double-positive for *AVP-Cre* and *Ai162-nCTG* (+/*AVP-Cre; +/nCTG*) received only rabies virus (RV) injection. In the AAV-based approach (AAVs), a mixture of AAVs expressing TVA and RG was injected into the PVH of *AVP-Cre* (+/*AVP-Cre; +/+*) mice prior to RV injection. (C) Representative coronal sections containing starter cells, defined as the overlap of mCherry (magenta) and GFP (green), in the PVH. Blue, DAPI. Scale bar, 30 μm. (D) The number of starter cells in the PVH was significantly larger in the conventional AAV-based approach (**p < 0.01, two-tailed Welch’s *t*-*t*est). (E) Representative coronal sections containing starter cells in the SO and SCH. Scale bar, 30 μm. (F) The number of starter cells in each region of  +/*AVP-Cre; +/nCTG* mice (**p < 0.01, one-way ANOVA with repeated measurements with post hoc Tukey’s HSD). (G) Representative coronal sections of presynaptic cells revealed by rabies-GFP. Abbreviations follow the Allen Mouse Brain Atlas [36]. Scale bars, 50 μm. (H) Normalized inputs from various nuclei to the PVH AVP neurons. The convergence index was defined as the number of labeled presynaptic neurons in each brain region normalized to the number of starter cells. The convergence index of *nCTG*-based approach was not significantly different from that of AAV-based approach (p > 0.34, two-way ANOVA). n = 6 and 7 mice for *nCTG* and AAVs, respectively. Error bars, SEM.

Recognizing these limitations, we next examined the performance of *nCTG*-mediated trans-synaptic tracing with RV, using the conventional AAV-based approach [11] as a control. The performance of these two methods can be comparable, as the TIGRE2.0 system can express reporter genes at levels similar to those achieved with AAV [15]. Although we did not examine the expression level of TVA and RG directly, at least the expression level of *nCTG*-mediated mCherry was within the range of AAV (Fig 2). We prepared *AVP-Cre; nCTG* female mice and injected RV into the unilateral PVH (Fig 3A and 3B). In the AAV-based approach, a mixture of two AAVs expressing either the TVA receptor or RG was injected into the unilateral PVH of *AVP-Cre* female mice, followed by RV injection (Fig 3A and 3B). In the PVH, we found that the number of starter cells, defined as an overlap of rabies-GFP and mCherry, was significantly higher in the AAV-based approach compared with *nCTG* (Fig 3C and 3D), presumably because of the lower targeting efficiency of *nCTG* and the reduced number of *AVP+* neurons (Fig 2). Unlike the AAV-based approach, *nCTG* may form second-order starter cells, i.e., if RV spreads from the PVH to *AVP-Cre*-positive cells in other brain regions, these cells can spread the virus to further presynaptic neurons as a result of RG expression. Therefore, we counted the number of these second-order starter cells in the supraoptic (SO) and suprachiasmatic (SCH) nuclei (Fig 3E). We confirmed that the number of starter cells in the SO and SCH was significantly smaller than that in the PVH (Fig 3F).

Both the AAV-based and *nCTG*-based approaches visualized presynaptic neurons of PVH AVP neurons in similar regions, consistent with previous studies using rabies virus-mediated tracing [20,21] (Fig 3G). We calculated the convergence index, defined as the number of labeled presynaptic neurons in each brain region normalized to the number of starter cells in the PVH. We found that the AAV-based approach exhibited denser labeling in all tested regions, although we did not find statistical significance between the AAV-based and *nCTG*-based approaches (Fig 3H). Collectively, these results suggest that although *nCTG*-mediated trans-synaptic tracing yields qualitatively comparable results to the AAV-based approach in identifying brain regions where presynaptic cells are distributed, it may not fully capture all neural connections that AAV-based tracing can label.

**Fig 4:**
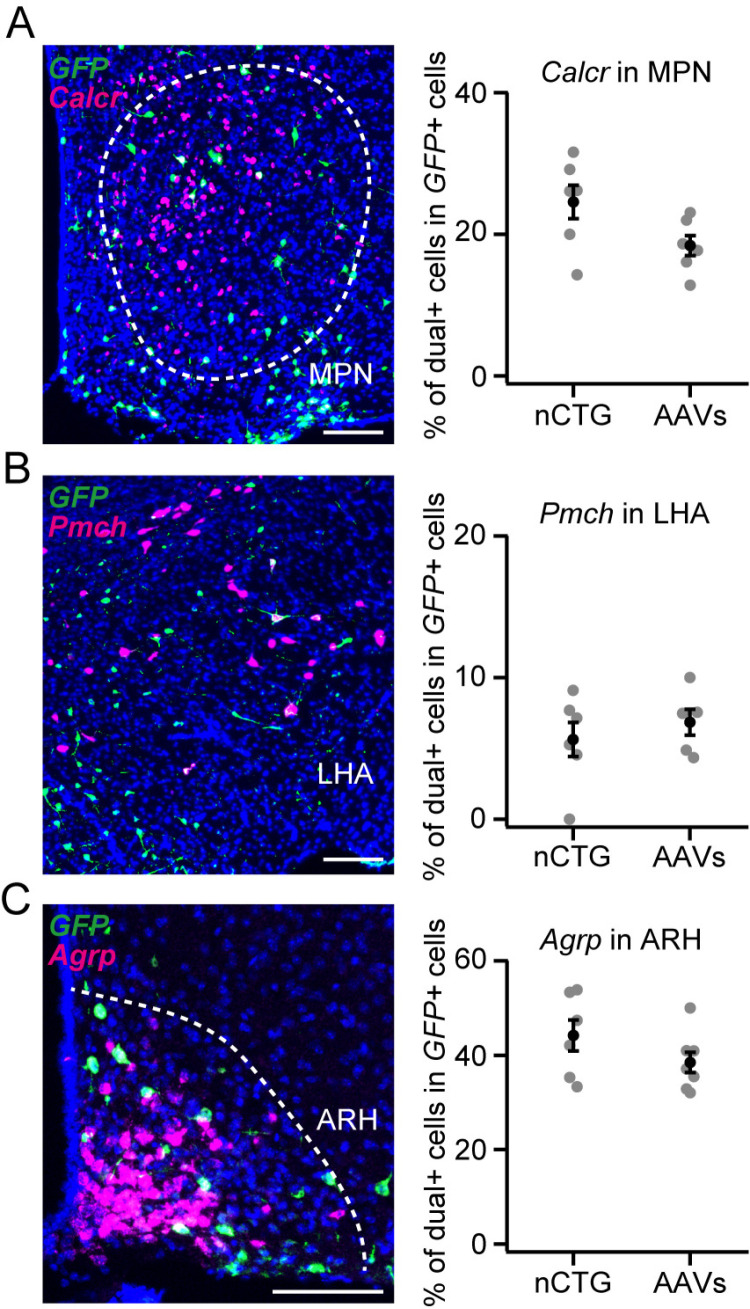
Cell-type characterization of presynaptic neurons by *in situ* hybridization. (A–C) Left, representative coronal sections showing *GFP* expression by rabies virus (green) and *Calcr* (A), *Pmch* (B), or *Agrp* (C) expression (magenta) using AAV-based approach. Blue, DAPI. Scale bars, 50 μm. Right, the fraction of *GFP*-positive cells co-expressing each marker gene. For *Calcr*, n = 6 each; for *Pmch*, n = 6 and 5 for nCTG and AAVs, respectively; for *Agrp*, n = 6 and 7 for nCTG and AAVs, respectively. No significant differences were observed (p = 0.09, 0.79, and 0.23 for *Calcr*, *Pmch*, and *Agrp*, respectively, by a two-tailed Mann–Whitney *U*-test). Error bars, SEM.

The lower convergence index exhibited in the *nCTG*-based approach may result from specific presynaptic populations remaining selectively unlabeled. Alternatively, *nCTG*-based tracing might exhibit an overall reduction in labeling efficiency without bias. To examine these possibilities, we performed *in situ* hybridization to identify the cell types of labeled presynaptic neurons. We visualized *calcitonin receptor* (*Calcr*) in the MPN [22,23], *pro-melanin concentrating hormone* (*Pmch*) in the lateral hypothalamic area (LHA) [24], and *agouti-related neuropeptide* (*Agrp*) in the arcuate hypothalamic area (ARH) [25] (Fig 4A–4C). We found that the fraction of cells expressing both *GFP* and these markers was indistinguishable between the *nCTG*- and AAV-based approaches (Fig 4A–4C). Collectively, these results suggest that *nCTG*-mediated trans-synaptic tracing misses a fraction of presynaptic inputs in an unbiased manner.

To exclude the possibility that the observed reduction in trans-synaptic tracing efficiency was due to a mutation in *RG*, we conducted PCR-based sequencing analysis of the entire transgene using primers flanking the knock-in cassette (Materials and Methods). This analysis confirmed that the *RG* sequence was completely intact, indicating that the reduced trans-synaptic tracing efficiency is not attributable to the RG cassette of *nCTG*. However, we unexpectedly identified mutations at the 5′ end of the *TVA* gene (see Materials and Methods for details), resulting in two amino acid substitutions—the first methionine was replaced by isoleucine, and the second alanine by threonine. Because the 2a-peptide cleaves immediately before the first methionine position, the former substitution would not affect the expression level of the TVA receptor. Although we have not directly assessed the impact of the latter substitution, which occurs close to the N-terminal extracellular region of the TVA receptor, the reduced number of starter cells in *nCTG* mice (Fig 3D) likely reflects the decreased number of PVH AVP neurons (Fig 2D). Therefore, we conclude that the TVA receptor with these two mutations likely functions similarly to wild-type TVA receptors, although we cannot entirely exclude the possibility that they may affect trans-synaptic tracing performance. Because these mutations were absent in the PITCh donor, they were likely introduced during the knock-in process, despite being distant from the CRISPR-mediated cleavage sites. This highlights a general precaution for CRISPR-based knock-in strategies: the entire knock-in cassette should be thoroughly validated by sequencing.

### Rabies virus-mediated trans-synaptic tracing during post-weaning development

**Fig 5:**
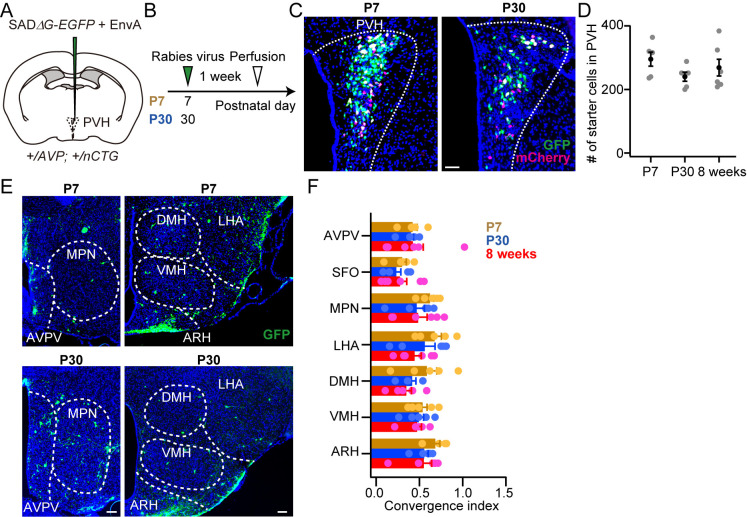
Comparison of input to PVH AVP neurons by injecting RV at P7, P30 and 8-week-old females. (A, B) Schematic of the virus injection. Female mice were used in this experiment. Rabies virus was injected into the unilateral PVH at postnatal day (P) 7, 30 or 8 weeks of age. Data for 8-week-old mice correspond to the “nCTG” data in Fig 3. (C) Representative coronal sections containing starter cells, defined as the overlap of mCherry (magenta) and GFP (green), in the PVH. Blue, DAPI. Scale bar, 30 μm. (D) The number of starter cells in the PVH did not differ significantly between the three groups (one-way ANOVA). n = 5, 5 and 6 mice for P7, P30 and 8 weeks groups, respectively. (E) Representative coronal sections of presynaptic cells revealed by rabies-GFP. Scale bars, 50 μm. (F) Normalized inputs from the nuclei to the PVH AVP neurons. In the brain regions that we examined, no significant differences were found between ages (p > 0.48, two-way ANOVA). Error bars, SEM.

Finally, we examined the presynaptic landscape of PVH AVP neurons during post-weaning development. We prepared *AVP-Cre; nCTG* females and injected RV at P7 or P30 (Fig 5A and 5B). We compared these data with corresponding data from 8-week-old females (Fig 3). The number of starter cells in the PVH was not significantly different (Fig 5C and 5D), suggesting that the number of PVH AVP neurons remained unchanged from P7, P30 to adulthood. We found that nCTG successfully visualized presynaptic neurons in P7 and P30 groups (Fig 5E). The convergence index between P7 and P30 groups were largely similar, yet we found a tendency to decrease in inputs from all nuclei we examined (Fig 5F). We also found that the convergence index remained largely similar between P30 and 8-week-old females (Fig 5F). These results suggest that the afferent neural connections to the PVH AVP neurons are largely invalid during pubertal development. Additionally, these data offer a proof-of-concept demonstration that *nCTG*-based trans-synaptic tracing can effectively map neural connections in neonatal and juvenile mice.

## Discussion

Rabies virus-mediated trans-synaptic tracing has revealed a number of neural connections and been instrumental in analyzing neural circuit formation. For example, researchers have described the development of vibrissal circuits [14] and the integration of newborn cells into existing networks in the adult dentate gyrus [26] and olfactory bulb [27]. A recent study in the mouse cortex utilized canine adenovirus type 2 (CAV-2)-mediated Cre expression to initiate trans-synaptic tracing to analyze the maturation of visual system circuits during adolescence [28]. Despite these successful tracings, describing neural inputs to specific cell types during postnatal development with single-nucleus resolution remains challenging. In the present study, we sought to address this requirement by generating a novel transgenic mouse line, *Ai162-nCTG*. Although our *Ai162-nCTG* line enabled the visualization of presynaptic neurons in neonatal or juvenile mice by RV, we found several experimental constraints. Here, we discuss the insights from our study and its limitations.

Our *Ai162-nCTG* line expresses nuc-mCherry, the TVA receptor, and RG in a Cre-dependent manner. Using the *AVP-Cre* line as an example, we showed that RV infected the Cre-expressing neurons and successfully labeled presynaptic neurons. However, the *Ai162-nCTG* line exhibited several drawbacks. First, progenies carrying both *Cre* and *nCTG* can be embryonically lethal in some Cre lines (Fig 1C). Although we did not observe this in the present study, premature death or stunted growth can also occur depending on the Cre driver [15]. These results suggest that not all Cre lines are suitable for experiments. Second, the targeting efficiency of *nCTG* expression is relatively low, preventing all Cre-expressing neurons from becoming starter cells (Fig 2E). Third, mice harboring both *Cre* and *nCTG* may exhibit a reduced number of Cre-expressing neurons. In the case of *AVP-Cre*, this phenomenon changed mouse behavior and physiology, such as increased urine volume and water consumption (Fig 2). Also, we cannot exclude the possibility that stronger expression of TVA and RG affects circuit formation, though we have shown that both the AAV-based and *nCTG*-based approaches visualized presynaptic neurons of PVH AVP neurons in similar regions (Fig 3). Fourth, although the *TIGRE2.0* system can express reporter genes at high levels, comparable to AAV [15], the convergence index of *nCTG*-based tracing was consistently lower than that of the AAV-based approach (Fig 3). This smaller convergence index suggests that the *nCTG*-based approach may fail to visualize some neural inputs to PVH AVP neurons.

Although these issues are difficult to address, we propose some potential solutions. The first issue likely arises from the *TIGRE2.0-tTA2* system rather than the inserted gene cassette in the *nCTG* line. For example, the original *Ai162* study [15] documented that double-positive progeny for some Cre driver and *Ai* lines can experience embryonic lethality, premature death, or stunted growth, presumably because of high tTA2 expression. To avoid such phenotypes, Cre drivers that label smaller neural populations can be used [15]. Alternatively, although we did not test this in the present study, bypassing embryonic transgene expression and activating Cre at appropriate developmental stages using tamoxifen-inducible CreER lines might prevent these phenotypes. Similarly, the second problem may also stem from the *TIGRE2.0* system, as the original study showed that a fraction of Cre-expressing neurons did not co-express the expected reporter [15]. The third and fourth issues might result from trade-offs regarding expression levels: strong expression might damage Cre-expressing cells, whereas lower expression of the TVA receptor and RG might reduce the efficacy and spread of RV infection. This trade-off is difficult to resolve with our current genetic approach. A new genetic scheme might be required to express both the TVA receptor and RG specifically and sufficiently. In addition, utilizing RG from a less-toxic rabies strain might mitigate the potential toxicity [29].

In this study, we performed rabies virus-mediated trans-synaptic tracing in PVH AVP neurons in P7, P30 and 8-week-old females (Fig 5). We found that the afferent neural connections to PVH AVP neurons were largely similar between these groups. Functionally, AVP secreted from the hypothalamus plays vital roles in behavior and physiology in rodents [30–32], with some functions being crucial for survival. This is supported by observations that mice with reduced AVP expression levels die within 1 week after birth [18,19]. Therefore, maintaining afferent neural connections that contribute to the activity of AVP neurons is likely essential. Investigating whether these afferent connections remain unchanged throughout life or adapt to life events is an interesting topic, considering that the pattern of presynaptic neurons to PVH oxytocin neurons changes when a virgin male becomes a father [33]. We propose that the *Ai162-nCTG* line, along with its future improvements, could be a useful tool for conducting some of these trans-synaptic tracing experiments.

## Materials and methods

### Ethics statements

All data presented in this study were obtained from experiments conducted in mice. All procedures described in the Materials and Methods were approved by the Institutional Animal Care and Use Committee of the RIKEN Kobe branch. In Fig 1, we monitored the health and behavior of the dams daily throughout their pregnancy, parturition, and the lactating period. After the birth of pups, we rarely found dead pups, suggesting that the deviation from the expected Mendelian ratio observed in some Cre drivers (Fig 1C) was because of embryonic lethality. For sacrifice, mice were deeply anesthetized with isoflurane and perfused with PBS, followed by 4% PFA in PBS.

### Animals

Animals were housed under a standard 12-h light/12-h dark cycle with *ad libitum* access to food and water. Wild-type C57BL/6J mice were purchased from Japan SLC, while *Ai162* (*TIT2L-GC6s-ICL-tTA2*, Jax #031562), *vGluT2-Cre* (also known as *Slc17a6-ires-Cre*, Jax #028863), *vGAT-Cre* (also known as *Slc32a1-ires-Cre*, Jax #028862), *OT-Cre* (Jax #024234), and *AVP-Cre* (Jax #23530) mice were purchased from the Jackson Laboratory. The *OTR-iCre* mouse line [34] (RIKEN BRC11687; Jax #037578) was provided by Drs. Yukiko U. Inoue and Takayoshi Inoue. The *ChAT-Cre* mouse line (Jax #006410) was a kind gift from Bradford Lowell.

### Generation of *Ai162-nCTG* mice

The *Ai162-nCTG* line (Accession No. CDB0135E; https://large.riken.jp/distribution/mutant-list.html) was generated by CRISPR/Cas9-mediated knock-in in zygotes as previously described [35]. The donor vector consisted of a nuclear localization signal, mCherry, TVA, and RG, which was generated for the CRISPR/Cas9-based PITCh system [16]. The mCherry with Kozak and a nuclear localization signal (*Kozak-nuc-mCherry*) and *F2A-TVA-T2A-RG* were obtained from pAAV-*CAG-FLEx-TCb* (Addgene #48332) [10] and pAAV-*TRE-HTG* (Addgene #27437) [11], respectively, with the following primers:

- *Kozak-nuc-mCherry*, forward, 5′-GCGAATTGGGTACCgtcgacGCCACCATGgctcctaagaagaagaggaaggtgGTGAG
- *Kozak-nuc-mCherry*, reverse, 5′-CTTGTACAGCTCGTCCATGC
- *F2A-TVA-T2A-RG*, forward, 5′-GACGAGCTGTACAAGggtagtggtgtgaaacagactttg
- *F2A-TVA-T2A-RG*, reverse, 5′-AAAGCTGGAGCTCgcggccgcttacagtctggtctcacccccactc

The guide RNA (gRNA) sites were designed to target upstream and downstream of *GCaMP6s*. The gRNA sites on the Ai162 allele were 5′-ATCACGCGTGCCGCCACCATGGG and 5′-CCACGTGATGACAAACCTTGGAG, where the last and first three bases are the protospacer adjacent motif sequence, respectively. Due to this gRNA design, 124 bases of the *GCaMP* gene remained. Microhomology-mediated end-joining resulted in the knock-in of *nuc-mCherry*, *TVA*, and *RG* (Fig 1A). For microinjection, a mixture of three crRNAs (CRISPR RNAs) (50 ng/mL), tracrRNA (trans-activating crRNA) (300 ng/mL), donor vector (10 ng/mL), and Cas9 protein (100 ng/mL) was injected into the pronucleus of *Ai162* one-cell stage zygotes. The crRNA and tracrRNA sequences were g1, 5′-AUC ACG CGU GCC GCC ACC Aug uuu uag agc uau gcu guu uug-3′; g2, 5′-CUC CAA GGU UUG UCA UCA CGg uuu uag agc uau gcu guu uug-3′; PITCh g3, 5′-GCA UCG UAC GCG UAC GUG UUg uuu uag agc uau gcu guu uug-3′; and tracrRNA, 5′- AAA CAG CAU AGC AAG UUA AAA UAA GGC UAG UCC GUU AUC AAC UUG AAA AAG UGG CAC CGA GUC GGU GCU-3′. The primers #1–#6 shown in Fig 1B were as follows:

#1, 5′-GAACGAGATCAGCAGCCTCTGTTCCAC
#2, 5′-CCATGTGCACCTTGAAGCGCATG
#3, 5′-CCGATGTGCACAATCAGGTCTCAGGAG
#4, 5′-ACCATCCCCATCGATGTCTGCTTCC
#5, 5′-GATAACATGGCCATCATCAAGGAG
#6, 5′-CCCATGGTCTTCTTCTGCATTAC

We obtained 77 F_0_ founder mice, 16 of which were *mCherry*-positive as identified by PCR (primers #5 and #6). Further PCR and sequencing confirmed that two of the 16 mice had the correct junction structures where the knock-in occurred (primers #1 and #2, #3 and #4). Finally, we selected one mouse line (Line 2) for *Ai162-nCTG*, as it exhibited proper knock-in of *mCherry*, *TVA*, and *RG* by PCR (primers #1 and #4).

For the sequence analysis, the PCR products from primer #1 and #4 were subcloned into the pCR Blunt II TOPO vector (Zero Blunt TOPO PCR Cloning Kit, Thermo Fisher). In addition to primer #1 and #4, sequencing was performed with the following five primers:

5′-TTGGTCACCTTCAGCTTGG
5′-ATCAAGTTGGACATCACCTC
5′-AAAGCGCATCCAAGGAGC
5′-ACAGATCCCTTCACTCGAG
5′-GTCAGTCAGAACTTGGAATGAG

We found that the *Ai162-nCTG* line contained two unexpected mutations in the *TVA* gene: instead of the expected 5′-AT*GG*CG, the sequence obtained was 5′-AT*TA*CG, leading to two amino acid substitutions at the beginning of TVA—Met to Ile and Ala to Thr. We confirmed that these mutations were absent in the PITCh donor. Therefore, these mutations were likely introduced during the knock-in process. Although the absence of the initial Met is typically deleterious, it posed no significant issue in this case, as the TVA receptor follows the 2a peptide self-cleavage signal. While we cannot exclude the possibility that these mutations changed some features of the TVA receptor, the functionality was likely retained because the EnvA-coated RV properly infected to the targeted neurons (Figs 3 and 5).

Genotyping of *nCTG* (Fig 1C) was performed with primer #5 and #6.

### Stereotactic injection

We used the Allen Mouse Brain Atlas [36] to target the PVH. The coordinates for P30 and 8-week-old mice were 0.8 mm posterior, 0.2 mm lateral, and 4.5 mm ventral from the bregma. In P7 mice, the following coordinates were used: 0.6 mm posterior, 0.2 mm lateral and 3.0 mm ventral from the bregma. Mice were anesthetized with 65 mg/kg ketamine (Daiichi Sankyo) and 13 mg/kg xylazine (X1251; Sigma-Aldrich) via intraperitoneal injection and head-fixed to stereotactic equipment (Narishige). The injection volume of viruses was 200 nL and the speed was 50 nL/min. After the viral injection, the animals were returned to their home cages.

### Trans-synaptic retrograde tracing

The following AAV vectors were generated by the University of North Carolina Vector Core using the corresponding plasmids [11]: AAV serotype 5 *CAG-FLEx-TVA-mCherry* (2.4 × 10^13^ gp/mL) and AAV serotype 8 *CAG-FLEx-RG* (1.0 × 10^12^ gp/mL). The RV used in this study, SAD*ΔG-EGFP*+EnvA, was prepared by following the protocol with viruses, cell lines, and materials as previously described [33,37]. The titer was estimated to be 3 × 10^9^ infectious particles per mL based on serial dilutions of the virus stock, followed by infection of the HEK293-TVA800 cell line (a gift from Ed Callaway). For trans-synaptic tracing with the conventional AAV-based approach, 200 nL of a 1:1 mixture of AAV5 *CAG-FLEx-TVA-mCherry* and AAV8 *CAG-FLEx-RG* was injected into the unilateral PVH. Two weeks after the AAV injection, 200 nL of SAD*ΔG-EGFP*+EnvA was injected into the same brain region to initiate trans*-*synaptic tracing. For *nCTG*-mediated trans-synaptic tracing, 200 nL of SAD*ΔG-EGFP*+EnvA was injected into the unilateral PVH without injection of AAVs. One week after RV injection, mice were sacrificed and perfused with PBS followed by 4% PFA in PBS. The brain was post-fixed with 4% PFA overnight. Twenty-μm coronal sections were collected, and every fourth section was subjected to cell counting. Brain images were acquired using an Olympus BX53 microscope equipped with a 10× (N.A. 0.4) objective lens. Cells were counted manually using the ImageJ Cell Counter plugin. Only mice with 50 or more starter cells were analyzed. Since we generated starter cells in unilaterally, we only analyzed and reported ipsilateral labeling. Because every fourth section was collected, the reported number of starter cells (Figs 3D, 3F, and 5D) was compensated (×4) from the measured value.

### Histochemistry

Mice were anesthetized with isoflurane and perfused with PBS followed by 4% PFA in PBS. The brain was post-fixed with 4% PFA overnight. Twenty-μm coronal brain sections were made using a cryostat (Leica). Given that we collected every second section to analyze the number of *AVP+* neurons, the reported number was compensated (×2) from the measured value (Fig 2D). Fluorescent *in situ* hybridization was performed as previously described [33,38]. The primers (5′ – 3′) used to produce RNA probes were as follows (the first one, forward primer, the second one, reverse primer):

*OT*, 5′-AAGGTCGGTCTGGGCCGGAGA; 5′-TAAGCCAAGCAGGCAGCAAGC
*AVP*, 5′-ACACAGTGCCCACCTATGCT; 5′-CTCTTGGGCAGTTCTGGAAG
*GFP*, 5′-ACGTAAACGGCCACAAGTTC; 5′-CTTGTACAGCTCGTCCATGC
*mCherry*, 5′- AAGGGCGAGGAGGATAACAT; 5′-CTTGTACAGCTCGTCCATGC
*Calcr* (part 1), 5′-CTGCTCCTAGTGAGCCCAAC; 5′-AGCAAGTGGGTTTCTGCACT
*Calcr* (part 2), 5′-TCCCAGGAGCTGACCATATC; 5′-TAGCAGCAAGCAAGAGGTCA
*Calcr* (part 3), 5′-TTGCCCTTGGGTGCTATCTA; 5′-AGCAGAAGCGTTTCACACAA
*Pmch*, 5′-TCCAATGCACTCTTGTTTGG; 5′-GCCAACATGGTCGGTAGACT
*Agrp*, 5′-CCCAAGAATGGACTGAGCAT; 5′-TGCGACTACAGAGGTTCGTG

For *Calcr*, a mixture of parts 1–3 was used. Fluoromount (K024; Diagnostic BioSystems) was used as the mounting medium. Brain images were acquired using an Olympus BX53 microscope equipped with a 10× (N.A. 0.4) objective lens. Signal-positive cells were counted manually using the ImageJ Cell Counter plugin.

### Measurement of water consumption and food intake

To evaluate water consumption, we measured the weight of a bottle containing 200 mL of water before placing it in the cage. After 24 h, we weighed the bottle again, and the decrease in weight was recorded as the daily water consumption. Food intake was measured as previously described [39]. In brief, we placed pre-weighed standard food pellets (50 g, MFG, Oriental Yeast, Shiga, Japan; 3.57 kcal/g) in the cage and reweighed them 24 h later. Food intake was reported to significant digits of 0.1 g.

### Measurement of the area covered by urine

The area covered by urine was measured following a previously described procedure [40]. In brief, each mouse was allowed to explore a fresh cage lined with a sheet of filter paper for 1 h. The urine-marking pattern was visualized using UV transillumination (E-BOX CX5; Vilber). The captured image was binarized by applying an automatic thresholding function in ImageJ. The area covered by urine was calculated by dividing the number of white pixels (urine-marked) by the total number of pixels in the image.

### Measurement of mean pixel intensity of RFP channel

In Fig 2B, 8-week-old female mice harboring both *AVP-Cre* and *Ai162-nCTG* were used. In Fig 2C, 8-week-old *AVP-Cre* female mice that received AAV5 *CAG-FLEx-TVA-mCherry* injection into the PVH were used. Two weeks after the AAV injection, mice were scarified. Mice were anesthetized with isoflurane and perfused with PBS followed by 4% PFA in PBS. The brain was post-fixed with 4% PFA overnight. Twenty-μm coronal brain sections were made using a cryostat. Those brain sections were mounted by Fluoromount without staining with antibodies. In each brain section, DAPI and RFP images were acquired using an Olympus BX53 microscope equipped with a 10× (N.A. 0.4) objective lens. Same optical intensity was used throughout the experiment. The brain images were loaded in ImageJ and a square ROI was set on the DAPI-positive cells. By setting the same ROI in the corresponding RFP image, mean pixel intensity of RFP channel was measured.

### Data analysis

All mean values are reported as the mean ± standard error of the mean (SEM). The statistical details of each experiment, including the statistical tests used, the exact value of n, and what n represents, are shown in each figure legend. The p-values are shown in each figure legend or panel; nonsignificant values are not noted.

## Supporting Information

S1 Fig The original image of gel electrophoresis shown in Fig 1B. Top, gel electrophoresis of primers #1 and #2, bottom, #3 and #4.
(TIF)

S2 Fig The original image of gel electrophoresis shown in Fig 1B. Primers #1 and #4 were used.
(TIF)

S3 Fig The original image of gel electrophoresis shown in Fig 1B. Primers #5 and #6 were used.
(TIF)
