# Supplementary figures and images for: A transgenic mouse line for rabies virus-mediated trans-synaptic tracing in the postnatal developing brain
Source: PLoS One. 2025 May 12;20(5):e0323629. doi: 10.1371/journal.pone.0323629 (PMC12068592; doi:10.1371/journal.pone.0323629)

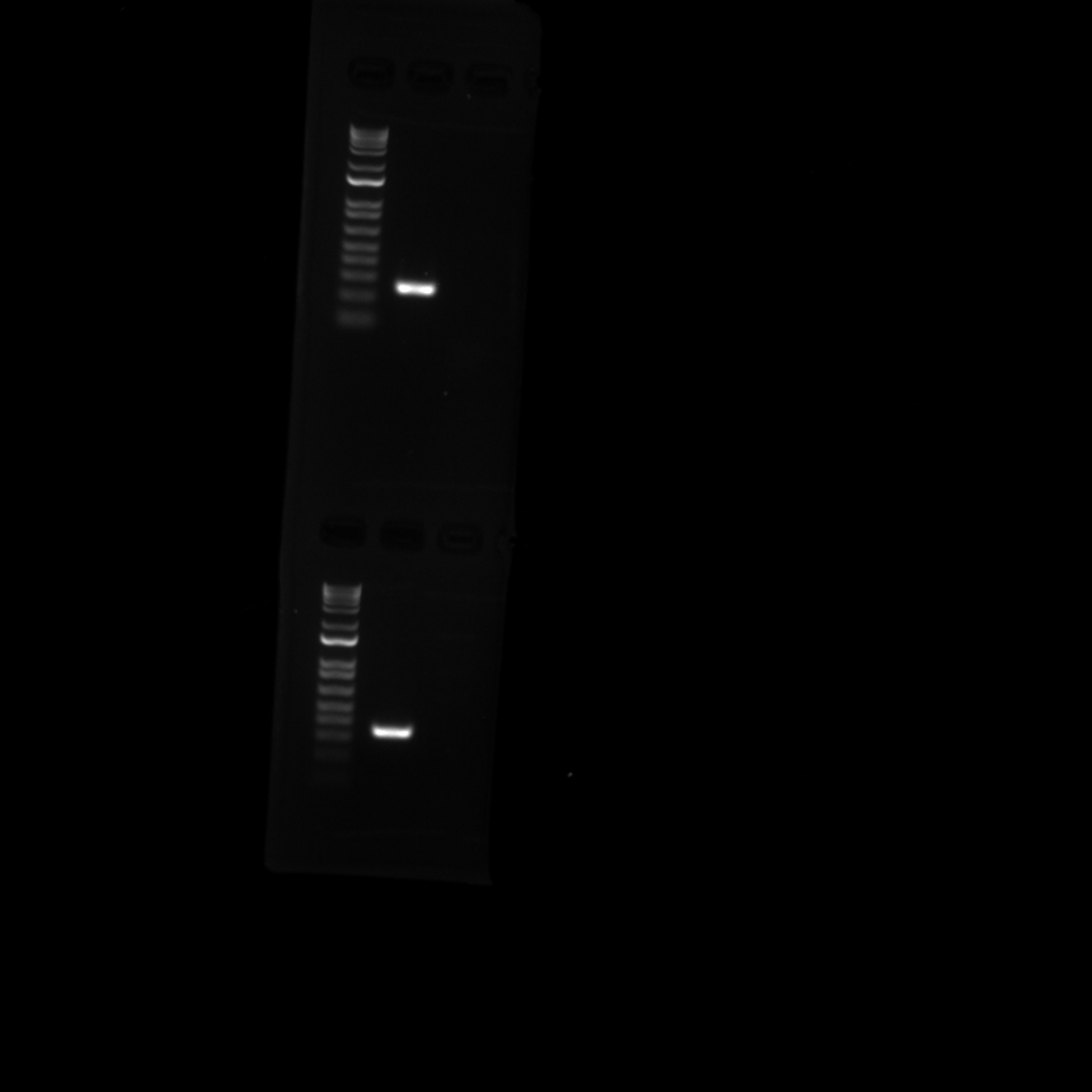

Supplement: S1 Fig — Top, gel electrophoresis of primers #1 and #2, bottom, #3 and #4. (TIF) [file pone.0323629.s001.tif]

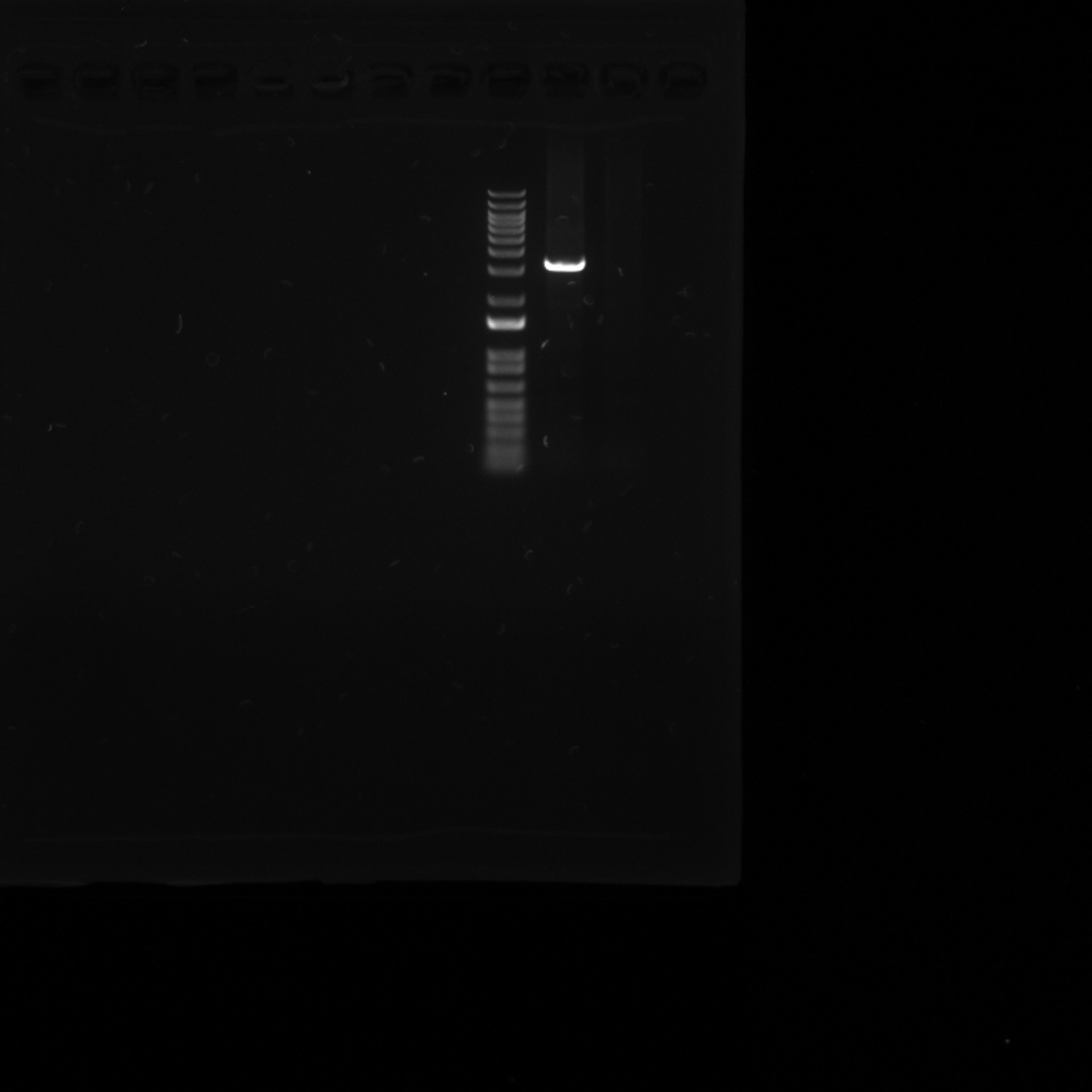

Supplement: S2 Fig — Primers #1 and #4 were used. (TIF) [file pone.0323629.s002.tif]

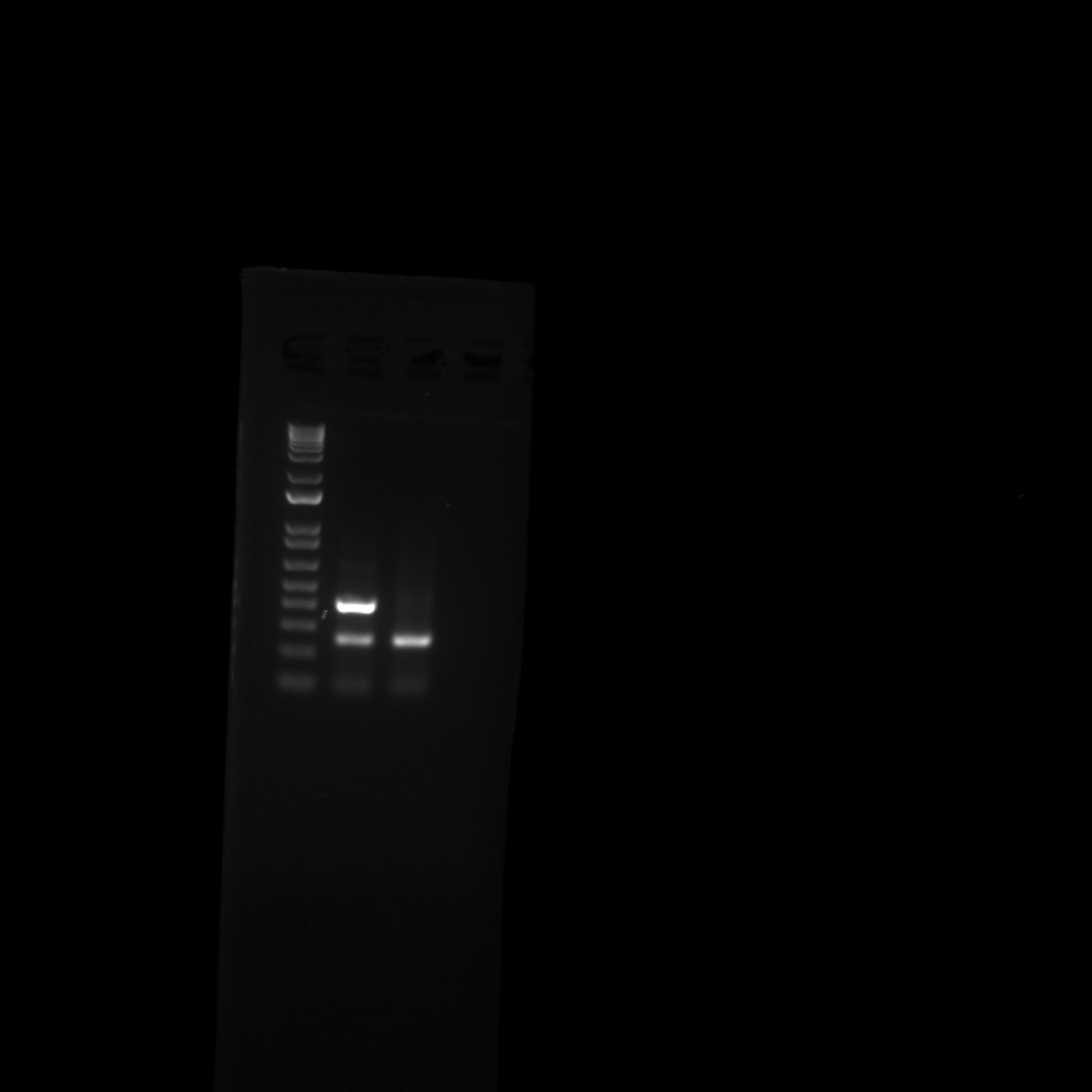

Supplement: S3 Fig — Primers #5 and #6 were used. (TIF) [file pone.0323629.s003.tif]
